# Analysis of the role of Arabidopsis class I *TCP* genes At*TCP7*, At*TCP8*, At*TCP22*, and At*TCP23* in leaf development

**DOI:** 10.3389/fpls.2013.00406

**Published:** 2013-10-16

**Authors:** José A. Aguilar-Martínez, Neelima Sinha

**Affiliations:** Department of Plant Biology, University of CaliforniaDavis, CA, USA

**Keywords:** Arabidopsis, TCP, transcription factor, leaf development, SRDX construct

## Abstract

TCP family of plant-specific transcription factors regulates plant form through control of cell proliferation and differentiation. This gene family is comprised of two groups, class I and class II. While the role of class II *TCP* genes in plant development is well known, data about the function of some class I *TCP* genes is lacking. We studied a group of phylogenetically related class I *TCP* genes: At*TCP7*, At*TCP8*, At*TCP22*, and At*TCP23*. The similar expression pattern in young growing leaves found for this group suggests similarity in gene function. Gene redundancy is characteristic in this group, as also seen in the class II *TCP* genes. We generated a pentuple mutant *tcp8 tcp15 tcp21 tcp22 tcp23* and show that loss of function of these genes results in changes in leaf developmental traits. We also determined that these factors are able to mutually interact in a yeast two-hybrid assay and regulate the expression of *KNOX1* genes. To circumvent the issue of genetic redundancy, dominant negative forms with SRDX repressor domain were used. Analysis of transgenic plants expressing AtTCP7-SRDX and AtTCP23-SRDX indicate a role of these factors in the control of cell proliferation.

## Introduction

Plant architecture and organ form rely on developmental processes which involve the control of cell proliferation, cell growth and cell differentiation (Ingram and Waites, 2006). TCP factors are involved in the coordination of cell proliferation and cell differentiation, having roles in several aspects of plant development, such as regulation of the shoot apical meristem (SAM) (Koyama et al., 2010; Li et al., 2012b), leaf development (Palatnik et al., 2003; Ori et al., 2007), and lateral branching (Doebley et al., 1997; Aguilar-Martínez et al., 2007).

TCPs are a plant-specific family of transcription factors, named after the transcription factors TEOSINTE BRANCHED1 (TB1) in maize, CYCLOIDEA (CYC) in *Antirrhinum majus*, and PCF1 and PCF2 (for PROLIFERATING CELL NUCLEAR ANTIGEN FACTOR1 and 2) in rice (Navaud et al., 2007; Busch and Zachgo, 2009).

*TCP* genes code for proteins with a 59-amino acid non-canonical basic helix-loop-helix (bHLH), the TCP domain, involved in DNA binding and dimerization (Cubas et al., 1999; Aggarwal et al., 2010). TCP proteins can be classified into two groups based on differences in the structure of the TCP domain, known as class I (or PCF or TCP-P) and class II (or TCP-C) [reviewed in Uberti Manassero et al. (2013)]. Mainly based on the TCP domain structure, class II TCP proteins can be subdivided into the CIN-like clade, with genes involved in lateral organ development such as *CINCINNATA* (*CIN*) in *Antirrhinum* (Nath et al., 2003), and the CYC/TB1 clade, with genes controlling axillary meristem development, such as *TB1* in maize (Doebley et al., 1997), and its orthologs in Arabidopsis (*Arabidopsis thaliana*) *BRANCHED1* (*BRC1*/*TCP18*) and *BRANCHED2* (*BRC2*/*TCP12*) (Aguilar-Martínez et al., 2007; Finlayson, 2007). Both class I and class II include members that can function as transcriptional activators and repressors (Uberti Manassero et al., 2013).

There are 24 predicted TCP proteins in the Arabidopsis genome, 13 class I and 11 class II proteins (Cubas, 2002). Functional analysis has been reported for most of the class II *TCP* genes. A subset of them, At*TCP2*, At*TCP3*, At*TCP4*, At*TCP10*, and At*TCP24*, are regulated by miR319a, produced by the *JAW* locus (Palatnik et al., 2003). In *jaw-D*, miR319a is overexpressed, which in turns down-regulates At*TCP2*, At*TCP3*, At*TCP4*, At*TCP10*, and At*TCP24*, resulting in plants which exhibit aberrant cell division, causing a serrated and crinkly leaf margin phenotype (Palatnik et al., 2003). On the contrary, forms of these five mRNAs that are resistant to the action of miR319a induce defects in the formation of a functional shoot meristem and cause fusion of cotyledons (Palatnik et al., 2003; Koyama et al., 2007; Nag et al., 2009). AtTCP3 is proposed to regulate the redundant *CUC* (for *CUP-SHAPED COTYLEDON*) genes, *CUC1* and *CUC2*, that function to delimit boundaries between lateral organs (Koyama et al., 2007). miRNA319-targeted TCPs interact with ASYMMETRIC LEAVES 2 (AS2) and regulate the expression of *KNOTTED1*-like *HOMEOBOX* (*KNOX*) genes *BREVIPEDICELLUS* (*BP*) and *KNAT2* (Li et al., 2012b). In tomato, a compound leaved species, *LANCEOLATE* (*LA*), an At*TCP4* homolog, controls compound leaf development. In the mutant *Lanceolate*, a miR319 resistant version of the *TCP* gene stimulates precocious cell differentiation, resulting in simple leaved plants (Ori et al., 2007). Mutant plants for *BRC1*/*AtTCP18* exhibit a loss of apical dominance while *tcp12* mutants show a milder phenotype (Aguilar-Martínez et al., 2007; Finlayson, 2007). BRC1 interacts with the florigen proteins FLOWERING LOCUS T (FT) and TWIN SISTER OF FT (TSF) to suppress the floral transition in axillary buds (Niwa et al., 2013). In case of At*TCP1*, the Arabidopsis homolog of *CYC*, the mutant *tcp1* shows no obvious phenotype (Cubas et al., 2001).

TCP proteins have been shown to mediate hormonal action in some instances. AtTCP1 regulates *DWARF4*, which produces an enzyme involved in brassinosteroid biosynthesis (Guo et al., 2010). AtTCP3 activates the expression of the auxin signaling repressor *IAA3/SHY2* (for *INDOLE-3-ACETIC ACID3/SHORT HYPOCOTYL2*, Koyama et al., 2010). AtTCP4 regulates the expression of the jasmonate biosynthesis gene *LIPOXYGENASE2* (Schommer et al., 2008) while the tomato *LANCEOLATE* acts to promote gibberellic acid biosynthesis (Yanai et al., 2011).

Much less is known about the function of class I TCP proteins in plant development. Of these, AtTCP21/CHE (for CCA1 HIKING EXPEDITION) is involved in the regulation of circadian clock activity (Pruneda-Paz et al., 2009). Also AtTCP15 and AtTCP11 interact with the core circadian component PRR5 (Giraud et al., 2010). AtTCP15 also regulates endoreduplication by modulating the expression of key cell-cycle genes (Li et al., 2012a). AtTCP16 is involved in pollen development (Takeda et al., 2006). AtTCP8 is found as one interactor factor with PNM1 (PPR protein to the nucleus and mitochondria 1), a potential coordinator of the expression of mitochondrial and nuclear genomes (Hammani et al., 2011). The expression of a repressor form of AtTCP11 (Viola et al., 2011) or AtTCP20 (Hervé et al., 2009) produces plants with pleiotropic developmental alterations. AtTCP14 regulates embryonic growth potential (Tatematsu et al., 2008; Rueda-Romero et al., 2012). AtTCP15, along with its closest homolog AtTCP14, has a role in internode length and leaf shape (Kieffer et al., 2011). Both AtTCP14 and AtTCP15 are found to interact with SPINDLY (SPY) to promote cytokinin responses (Steiner et al., 2012a) and increase the sensibility to this hormone when overexpressed in tomato (Steiner et al., 2012b).

Interplay between class I and class II TCP proteins has also been reported. Class I and class II proteins have distinct but overlapping binding sites: G(T/C)GGNCCC (Kosugi and Ohashi, 2002). Both classes may perform their functions by interacting with similar sets of target genes (Kosugi and Ohashi, 2002; Li et al., 2005). CIN-like TCP proteins regulate the expression of *CUC* genes through binding of the promoters of *miR164, ASYMMETRIC LEAVES1* (*AS1*), *IAA3/SHY2*, and the *SMALL AUXIN UP RNA* (*SAUR*) gene At1g29460 (Koyama et al., 2010), while AtTCP15, a class I TCP factor, also indirectly regulates *CUC* genes through binding the promoters of *IAA3/SHY2* and the *SAUR* gene At1g29460 (Uberti-Manassero et al., 2012).

Here we study the developmental role of class I TCP genes At*TCP7* (At5g23280), At*TCP8* (At1g58100), At*TCP22* (At1g72010), and At*TCP23* (At1g35560). AtTCP7, AtTCP8, AtTCP22, and AtTCP23 are expressed mainly in growing leaf tissues. Analysis of the pentuple mutant *tcp8 tcp15 tcp21 tcp22 tcp23* reveals the high level of genetic redundancy in the *TCP* gene family. These genes also interact with each other at the protein level. Plants with SRDX repression domain fusions for At*TCP7* and At*TCP23* show similar pleiotropic developmental alterations.

## Results

### Genetic redundancy is a characteristic of class I TCP factors

**Figure 1:**
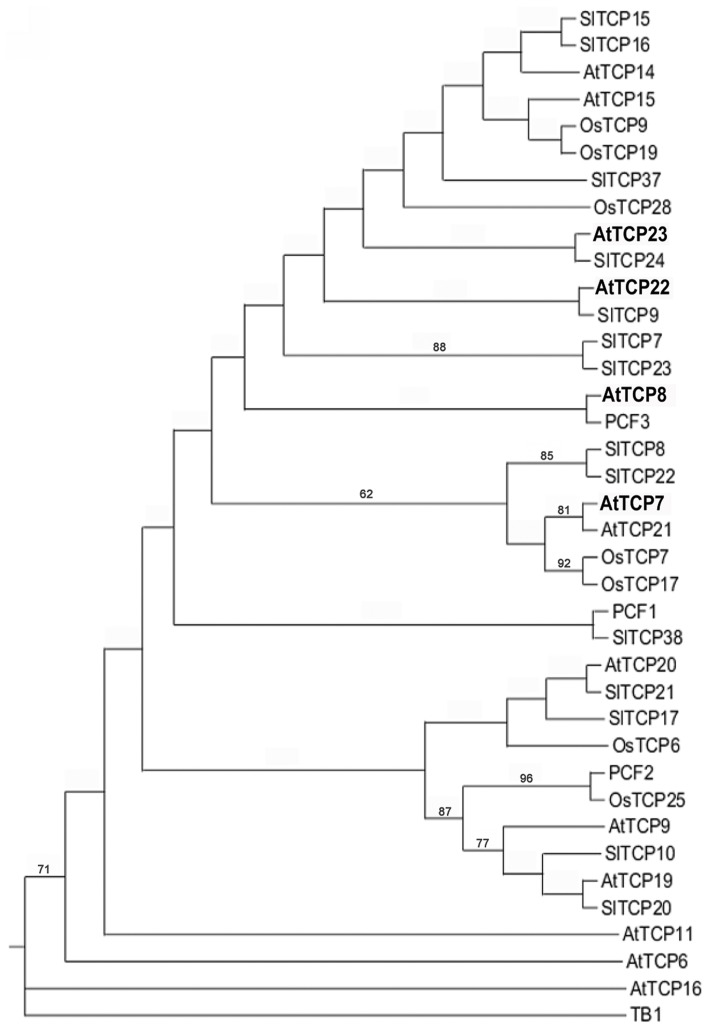
**Phylogenetic tree of class I *TCP* genes.** Phylogenetic tree of class I TCP proteins from Arabidopsis, tomato, and rice. Protein sequences were analyzed with the maximum-likelihood method by MEGA 5 software. Bootstrap values after 1000 replicates higher than 50% are shown. The tree was rooted using the translated sequence of the maize *teosinte-branched1* (*tb1*) gene as outgroup. In bold letters are the genes analyzed in this study.

Despite the described role of some TCP factors in plant development [reviewed in Uberti Manassero et al. (2013)] little is known about the function of a subset of class I TCP factors, AtTCP7, AtTCP8, AtTCP22, and AtTCP23. We determined the phylogenetic relationships of class I TCP factors using sequences from Arabidopsis, tomato, and rice (Figure 1 and Supplemental Figure 1). This analysis shows that AtTCP7, AtTCP8, AtTCP14, AtTCP15, AtTCP21, AtTCP22, and AtTCP23 form a distinct clade when compared to the rest of the factors in this subfamily such as AtTCP20, AtTCP9, AtTCP19, AtTCP11, AtTCP6, and AtTCP16. AtTCP7 and AtTCP21 are close homologs and show a 62% of sequence similarity at the protein level. AtTCP14 and AtTCP15 cluster together (Figure 1). Due to high sequence similarity, AtTCP8, AtTCP22, and AtTCP23 do not show clear phylogenetic relationships in this subgroup of class I TCP factors.

**Figure 2:**
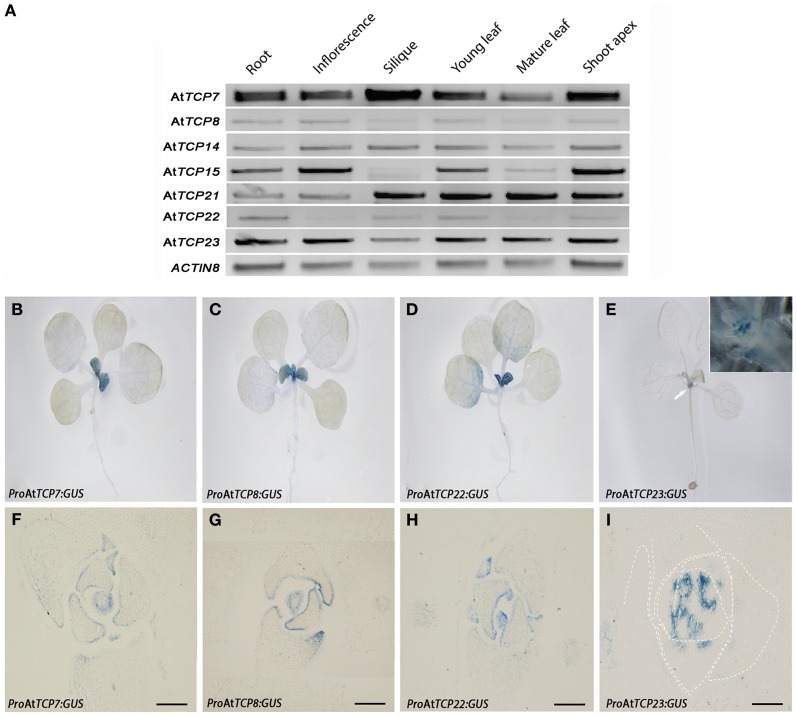
**Expression pattern of class I *TCP* genes. (A)** RT-PCR analysis of the tissue expression of class I *TCP* genes. *ACTIN8* was used as control gene. **(B–I)** Analysis of the expression pattern using the reporter marker *uidA* fused to the promoters of the genes in 12 days-old T3 transgenic plants. **(B)** *Pro*At*TCP7:GUS*; **(C)** *Pro*At*TCP8:GUS*; **(D)** *Pro*At*TCP22:GUS*; **(E)** *Pro*At*TCP23:GUS*. Inset, shoot apex, dashed lines indicate leaf primordia or leaf petiole margins. Arrow indicates the localization of the GUS signal. (**F–I)** Cross-sections of 12 days-old T3 lines. **(F)** *Pro*At*TCP7:GUS*; **(G)** *Pro*At*TCP8:GUS*; **(H)** *Pro*At*TCP22:GUS*; **(I)** *Pro*At*TCP23:GUS*. Dashed lines indicate leaf primordia. Bars = 100 μm.

We analyzed the expression pattern of class I *TCP* genes by RT-PCR in several tissues of Arabidopsis wild type plants (Figure 2A). At*TCP7*, At*TCP14*, At*TCP21*, and At*TCP23* are expressed in all the tissues analyzed. At*TCP15* and At*TCP8* are expressed at low levels in siliques and mature leaves, while At*TCP22* is not detected in inflorescences and mature leaves (Figure 2A). Expression data from Genevestigator (Hruz et al., 2008), the Arabidopsis eFP browser at BAR (Winter et al., 2007), AtGenExpress (Schmid et al., 2005), and GeneCAT (Mutwil et al., 2008) supports our RT-PCR analysis for At*TCP8*, At*TCP14*, At*TCP15*, At*TCP21*, and At*TCP23*, while for At*TCP7* and At*TCP22* there is no expression data yet available in these resources. Is interesting to note that according to this data At*TCP8* shows a high expression level in dry seeds.

To determine the spatial expression of class I TCP factors, we fused the complete 5′ gene promoter regions of At*TCP7*, At*TCP8*, At*TCP22*, and At*TCP23* to the coding sequence of the *uidA* marker gene and analyzed the GUS expression pattern in 12 days-old plants of T3 lines (Figures 2B–I). We found that the localization of GUS is restricted to young emerging leaves in plants *Pro*At*TCP7:GUS* (Figure 2B), *Pro*At*TCP8:GUS* (Figure 2C) and *Pro*At*TCP22:GUS* (Figure 2D). In plants *Pro*At*TCP23:GUS* the GUS signal is detected at the base of growing leaves (Figure 2E). Analysis of cross-sections showed that the GUS signal is located at the surface of the lateral organs and mainly in the adaxial side of the young leaves for plants *Pro*At*TCP7:GUS* (Figure 2F), *Pro*At*TCP8:GUS* (Figure 2G), and *Pro*At*TCP22:GUS* (Figure 2H). In plants *Pro*At*TCP23:GUS* the signal is found at the base of the growing leaves (Figure 2I). We did not detect signal at later stages of development in structures such as stem internodes, inflorescences, or flowers (data not shown). Thus, the class I TCP factors we studied have a similar expression pattern hinting at a similar function for these genes.

**Figure 3:**
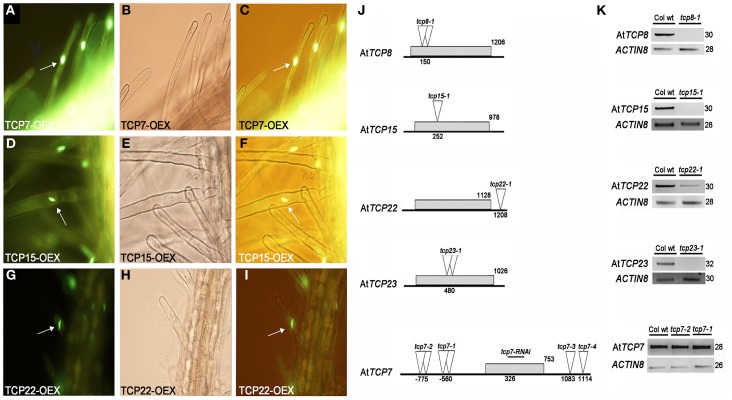
**Subcellular localization of class I TCP factors in root hair cells and characterization of class I *TCP alleles*. (A–C)** AtTCP7, **(D–F)** AtTCP15, **(G–I)** AtTCP22. **(A,D,G)** The GFP signal is located at the nuclei **(B,E,H)** Bright-field images. **(C,F,I)** Merged images. Arrows indicate nuclear location. **(J)** Scheme of the genes and position of the insertions. Gray boxes indicate the coding sequence of the genes. Triangles indicate the insertion lines. Double triangles indicate a tandem insertion. The fragment used for RNAi lines for *tcp7-RNAi* is indicated. Numbers indicate the position of the insertions or the length of the coding sequences respect to the start codon. **(K)** RT-PCR analysis of the alleles. *ACTIN8* was used as internal control. Number of cycles is indicated.

*TCP* genes are reported to code for a plant-specific family of transcription factors (Cubas et al., 1999). We analyzed the subcellular location of AtTCP7, AtTCP15, and AtTCP22 through fusions of the coding sequences of the corresponding genes to the marker GFP. We found that the fusion proteins are localized in the nucleus in case of AtTCP7 (Figures 3A–C), AtTCP15 (Figures 3D–F), and AtTCP22 (Figures 3G–I), suggesting a role of these TCP factors as transcription factors.

To determine the function of selected class I *TCP* genes, we characterized T-DNA insertion lines (Figure 3J). At*TCP7*, At*TCP8*, At*TCP15*, At*TCP22*, and At*TCP23* are intron-less genes. For At*TCP8*, At*TCP15*, and At*TCP23* we used the null alleles *tcp8-1, tcp15-1*, and *tcp23-1*, respectively (Figure 3K). For At*TCP22*, the allele *tcp22-1* showed a reduction in the level of the gene product (Figure 3K). In case of At*TCP7* we used four available insertion lines, however none of them was located in the coding sequence. RT-PCR expression analysis of the alleles *tcp7-1* and *tcp7-2* indicated that insertions outside the coding region do not lead to a reduction of transcript levels of At*TCP7* compared with the wild type (Figure 3K).

**Figure 4:**
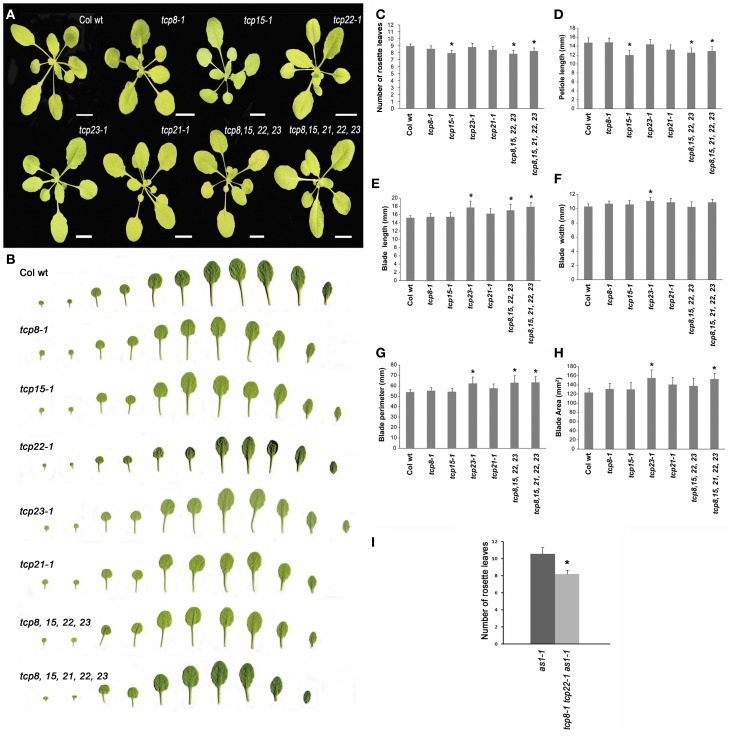
**Phenotypic characterization of class I *TCP* gene mutants. (A)** Rosettes at bolting stage of Col wt, the single mutants *tcp8-1, tcp15-1, tcp22-1, tcp23-1*, and *tcp21-1*, the quadruple mutant *tcp8-1 tcp15-1 tcp22-1 tcp23-1 (tcp8, 15, 22, 23)* and the pentuple mutant *tcp8-1 tcp15-1 tcp22-1 tcp23-1 tcp21-1 (tcp8, 15, 21, 22, 23)*. Bars are 1 cm. **(B)** Leaf series of plants at bolting stage of Col wt and the mutants *tcp8-1, tcp15-1, tcp22-1, tcp23-1, tcp21-1, tcp8, 15, 22, 23*, and *tcp8, 15, 22, 23, 21.* **(C–H)** Characterization of leaf traits in Col wt and class I *TCP* gene mutants. Number of rosette leaves **(C)**, petiole length **(D)**, blade length **(E)**, blade width **(F)**, blade perimeter **(G)**, and blade area **(H)**. **(I)** Number of rosette leaves in *AS1* allele *as1-1* and in the triple mutant *as1-1 tcp8-1 tcp22-1*. *n* = 13 to 28 in **(C–H)**; *n* = 30 to 33 in **(I)**. Asterisks (*) indicate statistically significant differences determined by Student's *t*-test, *p* < 0.05.

Phenotypically the single mutants are overall similar to the wild type (Figures 4A,B). We determined however that *tcp15-1* has reduced number of rosette leaves (Figure 4C). A quantitative analysis of leaf traits revealed that *tcp15-1* has shorter petiole length than wild type (Figure 4D). Kieffer et al. (2011) found a slightly shorter fruit pedicel length and shorter inflorescence height in this mutant. We also found that *tcp23-1* has increased blade length (Figure 4E), blade width (Figure 4F), blade perimeter (Figure 4G) and blade area (Figure 4H) compared to wild type. However we found that *tcp8-1* and *tcp21-1* plants are indistinguishable from wild type. For At*TCP7* we made RNAi lines (*tcp7-RNAi*) but the transgenic plants were similar to the wild type. As the analyzed single mutants only exhibited subtle phenotypic changes compared to the wild type we made multiple mutant combinations in the class I *TCP* genes reasoning that loss of function at multiple class I *TCP* genes would reveal more evident changes in plant development. We analyzed the quadruple mutant *tcp8-1 tcp15-1 tcp22-1 tcp23-1* and the pentuple mutant *tcp8-1 tcp15-1 tcp22-1 tcp23-1 tcp21-1*. The overt phenotype of both the quadruple and pentuple mutant was similar to the wild type (Figures 4A,B), although we found that these multiple mutants have fewer rosette leaves (Figure 4C) and bigger leaves than wild type (Figures 4E,G,H).

Mutations in *AS1* produce leaves with altered leaf shape, with the leaf lamina highly lobed (Byrne et al., 2000). CIN-like members of class II *TCP* genes also regulate leaf shape (Nath et al., 2003; Palatnik et al., 2003). Indeed, CIN-like TCP genes regulate the expression of *AS1* (Koyama et al., 2007). We wanted to determine if class I TCP mutations interact with the leaf shape phenotype of *as1*. We used the double mutant *tcp8 tcp22* to generate the triple mutant *as1 tcp8 tcp22*. We found that the number of rosette leaves produced in the triple mutant plants is reduced compared with the number of rosette leaves in *as1* while the leaf shape resemble the leaves of *as1* (Figure 4I), reinforcing the finding of class I *TCP* genes as regulators of leaf initiation rate.

In summary, this analysis indicates that single mutations in class I *TCP* genes have mild phenotypes. The quadruple and pentuple mutants have increased defects relating to number of leaves and leaf size while still maintaining normal morphology, indicating that other members in this subgroup of genes may act redundantly with the five genes analyzed.

### Class I TCP proteins can regulate the expression of cell-cycle and *KNOX1* genes and can interact with other members of the same class

**Figure 5:**
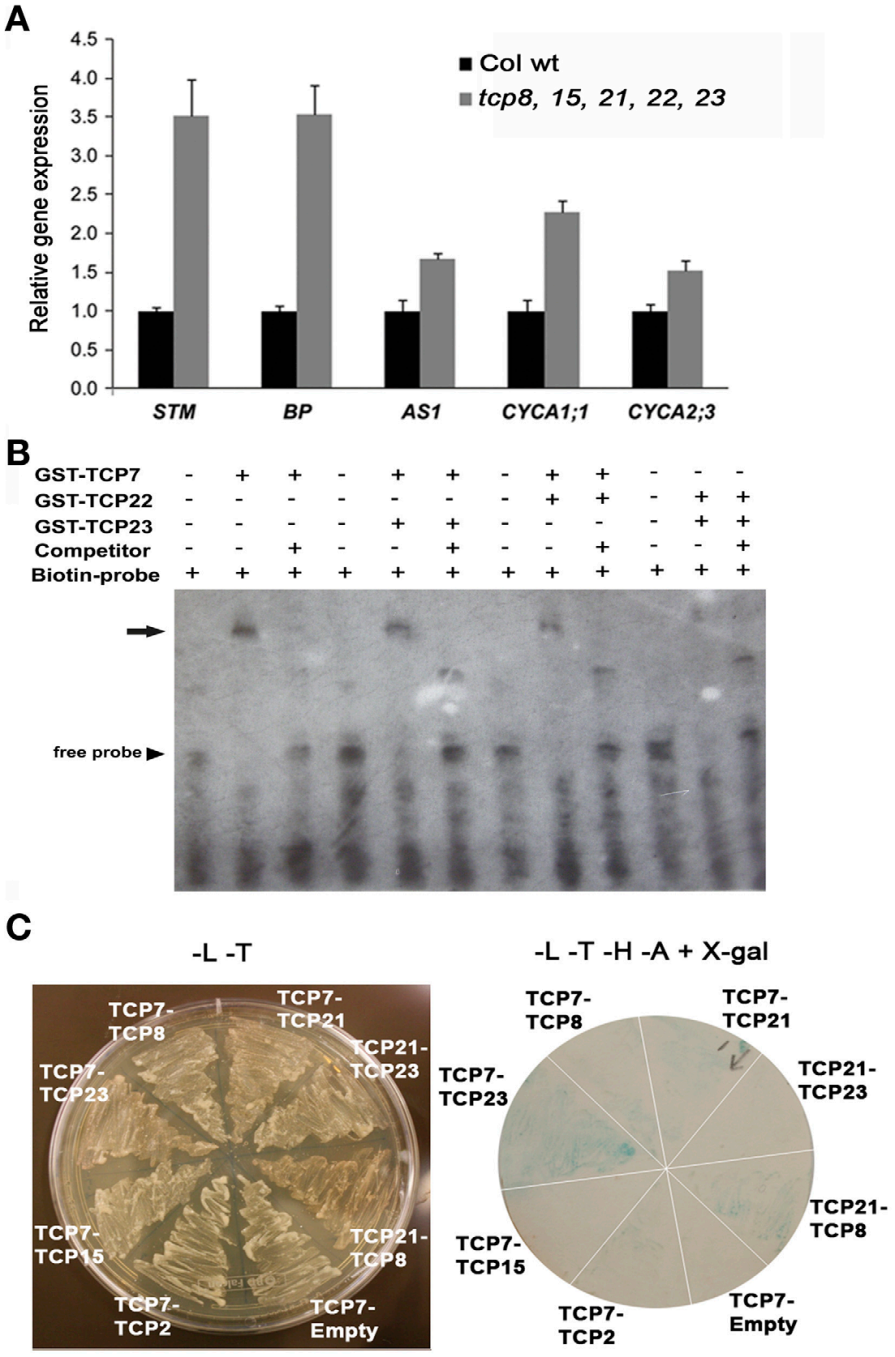
**Class I TCP factors regulate *KNOX1* genes and are capable of protein–protein interaction. (A)** Gene expression analysis by qPCR in Col wt and the pentuple mutant *tcp8, 15, 21, 22, 23*. Error bars are SD of three biological replicates. **(B)** EMSA of the K-box region of the *STM* promoter. Tested were factors AtTCP7, AtTCP22 and AtTCP23 in combinations indicated. Arrow indicates shifted bands. **(C)** Yeast-two hybrid assay to test for TCP protein–protein interactions. Left panel, co-transformed yeast grown in media without aminoacids Leucine and Tryptophan (-L-T media); right panel, β –Gal filter assay: co-transformed yeast were replica-plated in media without Leucine, Tryptophan, Histidine and Adenine and plus X-gal (-L-T-H-A+X-gal). The factors assayed were class I TCP factors AtTCP7, AtTCP8, AtTCP15, AtTCP21 and AtTCP23 and the class II factors AtTCP2 and AtTCP3. Interactions with empty vectors were used as negative controls.

It has been reported that some Arabidopsis class I *TCP* genes such as At*TCP20* (Li et al., 2005), At*TCP15* (Kieffer et al., 2011; Li et al., 2012a), and At*TCP14* (Kieffer et al., 2011) modulate the expression of cell-cycle genes. Furthermore, it has been shown that class I *TCP* genes can regulate the expression of genes involved in SAM and leaf development, acting in part redundantly with the class II *TCP* genes controlling the same set of genes (Uberti-Manassero et al., 2012). We wanted to determine if the expression of genes involved in cell-cycle control and SAM maintenance are affected in the pentuple *tcp* mutant *tcp8-1 tcp15-1 tcp22-1 tcp23-1 tcp21-1*. We found that genes involved in the control of SAM maintenance and leaf form such as the *KNOX1* genes *SHOOT-MERISTEMLESS* (*STM*) and *BP* and to a lesser extent *AS1* are overexpressed in the pentuple *tcp* mutant (Figure 5A). We also determined that genes regulating the cell-cycle such as *CYCA1;1* and *CYCA2;3* are up-regulated in the pentuple *tcp* mutant (Figure 5A). It is known that ectopic expression of *KNOX1* genes produces leaf lobing (Chuck et al., 1996). Although in the *tcp* pentuple mutant we found no dramatic leaf lobing alteration, the mutant plants have bigger leaf blades, and the up-regulation of cell-cycle genes can partially explain this increased leaf size.

The up-regulation of *KNOX1* genes in the *tcp* pentuple mutant indicates that class I TCP proteins could act directly or indirectly as regulators of *KNOX1* genes. We tested if class I *TCP* genes directly bind the *STM* promoter using electrophoretic mobility shift assay (EMSA). We used a probe of 76 bp that belongs to the K-box, a highly conserved region in the *STM* 5′-flanking sequence involved in the coordination of leaf development (Uchida et al., 2007). We found that AtTCP7 is capable to bind the K-box *in vitro* (Figure 5B). Mechanistically TCP factors can act as dimers and there is evidence that the DNA binding capacity is increased when they form heterodimers (Kosugi and Ohashi, 2002; Viola et al., 2011). We found that heterodimers AtTCP7-AtTCP23, AtTCP7-AtTCP22, and AtTCP22-AtTCP23 are also able to bind the K-box (Figure 5B).

This interaction between TCP factors can account for the high degree of genetic redundancy found in this family. We wanted to analyze if members of class I *TCP* genes analyzed in this study are able of protein–protein interaction using a yeast two-hybrid assay (Figure 5C and Table 1). We found that AtTCP7 interacts with AtTCP23, AtTCP21, and to a lesser extent with AtTCP8. We also found interaction between AtTCP8 and AtTCP21 (Figure 5C and Table 1). Class II TCP factors AtTCP2 and AtTCP3 do not show interaction with any of the class I TCP factors tested. Taken together these results indicate that class I TCP factors can have a similar gene function by virtue of their ability to form higher order complexes to regulate similar downstream targets.

**Table 1:** **Protein–protein interactions between TCP factors using yeast-two hybrid**.

	**pGBKT7-**						
	
	**TCP7**	**TCP8**	**TCP15**	**TCP21**	**TCP23**	**TCP2**	**Empty**
pGADT7-TCP7		+	−	+	+++	−	−
pGADT7-TCP8			−	++	−	−	−
pGADT7-TCP15						−	−
pGADT7-TCP21			−		−	−	−
pGADT7-TCP23			−			−	−
pGADT7-TCP2							−
pGADT7-TCP3		−	−	−	−		−
Empty							−

Signs are for strong (+++), medium (++), mild (+) and no interaction (−), respectively.

### Dominant negative forms of class I *TCP* genes affect lateral organ growth

**Figure 6:**
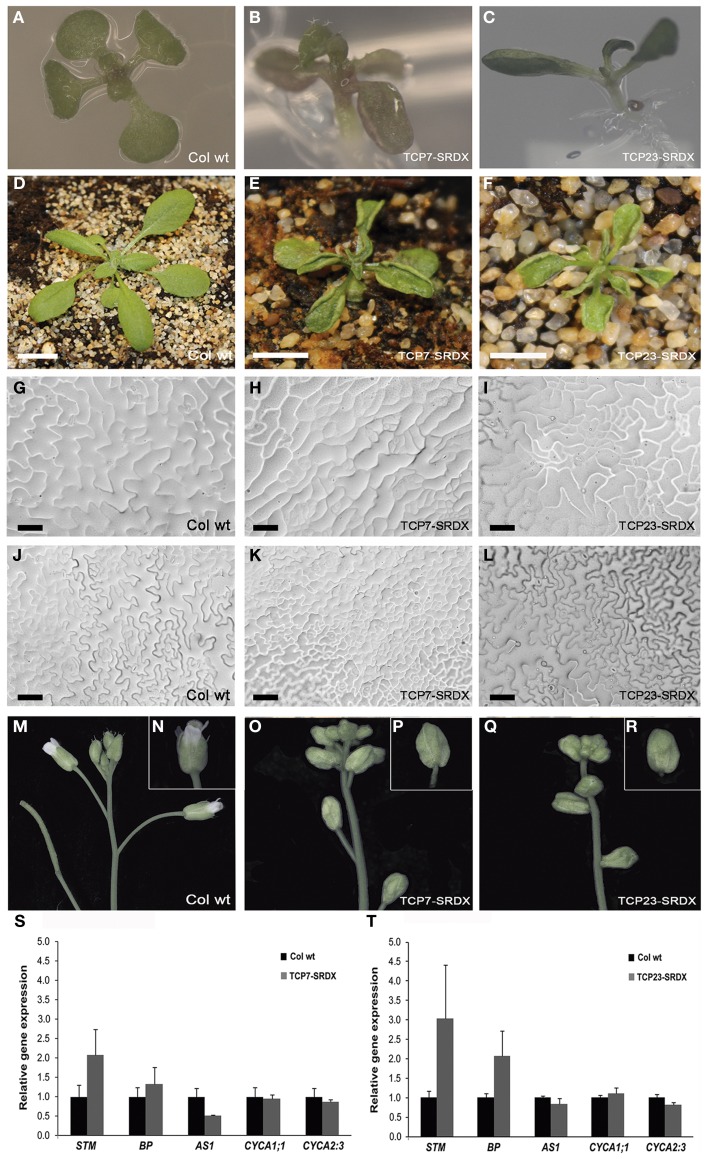
**Characterization of transgenic plants harboring class I *TCP* genes At*TCP7* and At*TCP23* fused to the repressor domain SRDX. (A–C)** Phenotype of 10 days-old seedlings of TCP7-SRDX and TCP23-SRDX transgenic lines. **(A)** Col wt. **(B)** TCP7-SRDX. **(C)** TCP23-SRDX. **(D)** Plants at bolting stage of Col wt; **(E)** of TCP7-SRDX and **(F)** of TCP23-SRDX. **(G–L)** Leaf epidermis nail polish impressions in Col wt **(G,J)**, TCP7-SRDX **(H,K)** and TCP23-SRDX **(I,L)** plants. **(G–I)** Adaxial side; **(J–L)** abaxial side. **(M)** Inflorescence and **(N)** isolated flower of a Col wt plant. **(O)** Inflorescence and **(P)** flower of a TCP7-SRDX transgenic plant. **(Q)** Inflorescence and **(R)** flower in a TCP23-SRDX transgenic plant. **(S)** qPCR expression analysis of some gene markers comparing Col wt plants with TCP7-SRDX plants and **(T)** with TCP23-SRDX plants. Error bars are SD of three biological replicates. Scale bars are: **(D)**, 1 cm; **(E), (F)**, 0.5 cm; **(G–L)**, 50 μm.

The lack of a severe phenotype in the pentuple mutant can be ascribed to genetic redundancy, particularly prominent in the *TCP* gene family (Schommer et al., 2008; Koyama et al., 2010). One way to circumvent genetic redundancy and to analyze the function of transcription factors is to transform them into dominant negative forms by fusing the coding sequences of the genes to the repressor domain SRDX, producing loss-of-function phenotypes even in the presence of redundant genes (Hiratsu et al., 2003). To further analyze the function of class I *TCP* genes, we generated transgenic lines with fusions of the coding sequences of At*TCP7* and At*TCP23* to the SRDX repressor domain and compared their phenotypes with the wild type (Figure 6). At the seedling stage lateral organs (cotyledons and first true leaves) of TCP7-SRDX and TCP23-SRDX plants show an upward growth (Figures 6A–C). At the bolting stage compared to the wild type (Figure 6A), TCP7-SRDX and TCP23-SRDX rosette leaves are hyponastically curved and smaller (Figures 6E,F). The basis of the differences in leaf size and form can be due to an altered cell growth pattern. We therefore analyzed the leaf epidermis and found that the leaves of the transgenic lines have groups of small cells on both adaxial (Figures 6G–I) and abaxial (Figures 6J–L) surfaces. Flowers are also affected in the transgenic plants (Figures 6M–R). Compared with the wild type (Figures 6M,N) the flowers of TCP7-SRDX (Figures 6O,P) and TCP23-SRDX (Figures 6Q,R) plants have shorter pedicels and are closed, with irregularly shaped larger sepals, shorter petals and less trichomes. Despite these alterations, the flowers have a normal number of sepals, petals, stamens, and carpels (data not shown). Gene expression analysis through qPCR on SAM gene markers indicated that *STM* is up-regulated in TCP7-SRDX (Figure 6S) and TCP23-SRDX (Figure 6T) transgenic plants, while the expression of the cell-cycle marker *CYCA1;1* is not changed and the expression of *CYCA2;3* is down-regulated (Figures 6S,T). In summary, dominant negative forms of At*TCP7* and At*TCP23* produce similar phenotypes with alterations in lateral organ growth, possibly based in differences in cell proliferation. Therefore is likely that At*TCP7* and At*TCP23* both function in regulating cell proliferation.

In conclusion, our results expand our knowledge of a group of members of the *TCP* gene family which has important roles in plant development. The lack of a severe phenotype in multiple mutants, the phenotype of the leaf epidermis and leaf shape found in dominant negative forms in these genes, their expression in young leaves, the demonstrated protein-protein interaction between selected members, the altered *KNOX1* gene expression in the knockout or dominant negative mutations, indicates that they may function in the control of leaf development by regulating similar gene networks.

## Discussion

The phylogenetic analysis showed that AtTCP14, AtTCP15, AtTCP23, AtTCP22, AtTCP8, AtTCP7, and AtTCP21 form a subgroup within the class I of TCP factors, AtTCP20, AtTCP9, and AtTCP19 form another clade while AtTCP11, AtTCP6, and AtTCP16 are more distantly related. The phylogenetic relationships of these factors can account for a related function. At*TCP11* and At*TCP16* have a role in pollen development (Takeda et al., 2006; Viola et al., 2011) and At*TCP9* and At*TCP20* act regulating the expression of the jasmonic acid biosynthesis gene *LOX2* (Danisman et al., 2012). At*TCP7* and At*TCP21* are close homologs. This raises the question if these two genes share a similar function. AtTCP21 (CHE) has a role in the control of the circadian rhythm, interacting with the core clock component TOC1 (for TIMING OF CAB EXPRESSION1). Giraud et al. (2010) analyzed the interaction of all the TCP factors with known components of the circadian clock. Interestingly they found that class I TCP factors AtTCP15 and AtTCP11 interact with TOC1 and other components of the core circadian clock, however they found no interactions in case of AtTCP21 or AtTCP7. AtTCP3, a member of the class II TCP factors, was also found to interact with TOC1 (Giraud et al., 2010). Moreover, another component of the core clock, CCA1 (for CIRCADIAN CLOCK ASSOCIATED1), binds to the promoter of *CHE* (Pruneda-Paz et al., 2009), however the promoter of At*TCP7* lacks this CCA-1 binding site. Furthermore, the expression pattern of these genes appears to be different. Using promoter reporter fusions to GUS it was found that At*TCP21* is expressed ubiquitously throughout the plant (Pruneda-Paz et al., 2009) while our data indicate that the expression of At*TCP7* is more restricted to growing new leaves. Taken together is possible that these genes have different functions.

Weak phenotypic alteration or absence of such alteration in single mutants of class II *TCP* genes is attributed to genetic redundancy (Schommer et al., 2008; Koyama et al., 2010). Mild or no phenotypes are present in loss of function of class I *TCP* genes such as At*TCP21* (Pruneda-Paz et al., 2009), At*TCP14* (Kieffer et al., 2011), At*TCP15* (Kieffer et al., 2011; Li et al., 2012a; Uberti-Manassero et al., 2012) and in this study for At*TCP8* and At*TCP23*. In this case the analysis of multiple mutants is useful to analyze the function of these genes. Other multiple mutants have been developed in the *CIN*-like clade of *TCP* genes. Koyama et al. (2010) developed the triple mutant *tcp3 tcp4 tcp10*, the quadruple mutant *tcp3 tcp4 tcp5 tcp10* and the pentuple mutant *tcp3 tcp4 tcp5 tcp10 tcp13*. These mutants show dose-dependent changes mainly in leaf morphology, however our analysis shows that the pentuple mutant *tcp8 tcp15 tcp21 tcp22 tcp23* has no dramatic differences compared with the wild type. This can indicate that analysis of higher order mutants are needed to more understand the function of these genes. For example in the analysis of the gene family of 9 members *LONELY GUY (LOG)* of cytokinin-activating enzymes, the sexptuple mutants *log1 log2 log3 log4 log5 log8* or *log1 log2 log3 log4 log5 log7* show no visible phenotype, however the septuple mutant *log1 log2 log3 log4 log5 log7 log8* show a extremely severe retardation of shoot and root growth with defects in the maintenance of the apical meristems (Tokunaga et al., 2012). At*TCP7* could not be included in our multiple mutant analysis due to a lack of an available T-DNA knockout insertion line. Further, *tcp22-1* is not a null allele and still retains some gene expression. The gene function of these two genes or another class I *TCP* genes such as At*TCP14* can be sufficient for normal plant growth. Future studies on the role of class I *TCP* genes would have to focus on multiple mutants such as the pentuple mutant *tcp8 tcp14 tcp15 tcp22 tcp23*, the sextuple mutant *tcp8 tcp14 tcp15 tcp21 tcp22 tcp23* or higher order multiple mutants.

Class II TCP factors and AS2 interact to directly regulate the expression of *KNOX1* genes (Li et al., 2012b). Due to the interplay between the two classes of *TCP* genes in regulating the expression of downstream genes (Danisman et al., 2012; Uberti-Manassero et al., 2012) it is tempting to argue that class I TCP factors can also regulate *KNOX1* gene expression. Our data indicates that *KNOX1* gene expression is altered in the pentuple mutant *tcp8 tcp15 tcp21 tcp22 tcp23*. *In vitro* studies indicate that class I TCP factors can bind the promoter of *STM*, a *KNOX1* gene. Furthermore, yeast-two hybrid experiments show that class I TCP factors are able to interact, as we found for AtTCP7 with AtTCP23, and AtTCP8 with AtTCP21. Heterodimerization improves the action of TCP factors, as reported by Kosugi and Ohashi (2002). Viola et al. (2011) showed interaction between the class I TCP factors AtTCP11 and AtTCP15 and Danisman et al. (2012) reported that AtTCP20 interacts with AtTCP8 and AtTCP22. TCP factors have been recognized as intrinsically disordered proteins (IDPs) and can also form multimers and interact with numerous other proteins (Valsecchi et al., 2013), which can account for the activity of these factors in multiple pathways (Babu et al., 2011; Valsecchi et al., 2013).

There is an interplay between *AS1-AS2, TCP*, and *KNOX1* genes. AS1-AS2 form a complex (Xu et al., 2003) able to bind the promoters of *KNOX1* genes *BP* and *KNAT2* (Guo et al., 2008). Class II TCP factors bind the promoter of *AS1* (Koyama et al., 2010). Class II TCP factors interact with AS2 (Li et al., 2012b) and are able to bind the promoters of *KNOX1* genes *BP* and *KNAT2* (Li et al., 2012b). We have determined that class I TCP factors are able to bind the promoter of *KNOX1* gene *STM*. We have also determined that class I TCP mutants affect the leaf initiation rate. This phenotype is enhanced in an *as1* mutant background, that indicates that *AS1* could have a role in the control of leaf initiation rate. Leaf initiation rate is influenced by cell division rate (Itoh et al., 1998; Wang et al., 2008) and *TCP* genes have a role in the control of cell division (Li et al., 2005). The number of rosette leaves is correlated with flowering time in natural accessions (Alonso-Blanco et al., 1998). However Salomé et al. (2011) indicate that flowering time and number of leaves can be genetically uncoupled. Recently it has been reported that the *TCP* gene *BRC1* interacts with flowering genes *FT* and *TSF* (Niwa et al., 2013). It can be interesting to determine if class I *TCP* genes interact with flowering genes and AS1-AS2 factors.

The generation of chimeric repressor mutants with the SRDX domain has been used to analyze the function of a number of genes, such as *TCP* genes including At*TCP1* (Guo et al., 2010), At*TCP3*, At*TCP2*, At*TCP4*, At*TCP5*, At*TCP10*, At*TCP13*, At*TCP17*, At*TCP24* (Koyama et al., 2007), At*TCP11* (Viola et al., 2011), At*TCP14* (Kieffer et al., 2011), At*TCP15* (Kieffer et al., 2011; Li et al., 2012a; Uberti-Manassero et al., 2012), and At*TCP20* (Hervé et al., 2009). In this study we fused the repressor domain SRDX to the coding sequences of At*TCP7* and At*TCP23*. Both types of transgenic plants show similar phenotypes, mainly characterized by an upward growth of the leaves and smaller rosette size. The similarity of phenotypes in AtTCP7-SRDX and AtTCP23-SRDX lines indicates that they likely have similar functions. Koyama et al. (2007) found similar phenotypes in SRDX constructs for the TCP genes At*TCP2*, At*TCP3*, At*TCP4*, At*TCP5*, At*TCP10*, At*TCP13*, At*TCP17*, and At*TCP24*, showing that all of them act redundantly as regulators of the expression of boundary-specific genes.

The phenotype of AtTCP7-SRDX and AtTCP23-SRDX plants is not similar to that seen for SRDX constructs of the class II *TCP* genes mentioned above (Koyama et al., 2007); instead it resembles to a certain extent the phenotype of plants expressing AtTCP15-SRDX (Uberti-Manassero et al., 2012) and AtTCP11-SRDX (Viola et al., 2011), in particular the upward leaf growth. However the phenotype of the inflorescences differs markedly, while flowers of AtTCP15-SRDX show exposed gynoecia (Kieffer et al., 2011; Uberti-Manassero et al., 2012), the flowers of AtTCP7-SRDX and AtTCP23-SRDX are closed. Is interesting to note that the phenotype of AtTCP15-SRDX described is with the use of the endogenous promoter of At*TCP15* (Kieffer et al., 2011; Uberti-Manassero et al., 2012), while the phenotype of AtTCP7-SRDX and AtTCP23-SRDX plants is with the use of a constitutive promoter. AtTCP15-SRDX plants under a constitutive promoter can produce plants with a very strong phenotype with SAM arrest (Uberti-Manassero et al., 2012), phenotype not found in AtTCP7-SRDX and AtTCP23-SRDX plants.

The role of TCP factors in the control of cell division arrest and leaf development is well documented (Nath et al., 2003; Ori et al., 2007). Other members of the class I TCP factors such as the rice PCF1 and PCF2 (Kosugi and Ohashi, 1997), AtTCP20 (Hervé et al., 2009) and AtTCP14 and AtTCP15 (Kieffer et al., 2011; Li et al., 2012a) have a documented role in the control of cell proliferation. Our analysis indicates that the cell-cycle marker *CYCA2;3* is down-regulated in AtTCP7-SRDX and AtTCP23-SRDX plants. Li et al. (2012a) also found that this marker is down-regulated. The leaf phenotype of AtTCP7-SRDX and AtTCP23-SRDX plants, the altered epidermal cell patterning, and altered cell-cycle gene expression suggest that class I *TCP* genes analyzed in this study can also regulate cell growth and leaf development by acting on cell-cycle genes.

The class I *TCP* genes analyzed in this study show a similar expression pattern, with the exception of At*TCP23*. We used the complete 5′ promoter region for the study of the spatial expression pattern. However the actual expression of At*TCP23* could require regulatory regions in other regions of the gene. It is likely that the encoded proteins retain similar functions as the plants with the constructs AtTCP7-SRDX and AtTCP23-SRDX have similar phenotypes.

## Materials and methods

### Plant materials and growth conditions

Alleles *tcp8-1* (SAIL_656_F11), *tcp7-2* (SAIL_180_B09), and *tcp23-1* (SAIL_443_F02) were obtained from the Syngenta Arabidopsis Insertion Library (SAIL) (Sessions et al., 2002). Alleles *tcp15-1* (SALK_011491C) and *tcp22-1* (SALK_027490) were obtained from the Salk Institute Genomic Analysis Laboratory (SIGnAL) (Alonso et al., 2003) through the Arabidopsis Biological Resource Center (ABRC). The mutant allele for At*TCP21* was *che-1* (Pruneda-Paz et al., 2009). The allele *tcp7-4* (WISCDSLOX240D10) was obtained from the Wisconsin DsLox collection (Woody et al., 2007). *tcp7-1* (GK-511D02) belongs to the GABI-Kat collection (Li et al., 2007). The gene allele *tcp7-3* was obtained from the INRA-Versailles T-DNA collection (Beschtold et al., 1993). *tcp7-3* is in Wassilewskija (Ws) background, all the rest are in Columbia-0 (Col-0) ecotype background. *as1-1* was from Uchida et al. (2007).

Plants were grown under long-day conditions (16 h light/8 h dark) at 22°C in a soil mixture (Sunshine Mix1). For the phenotypic analysis plants were grown in chambers at 23°C at 50% humidity and 60–70 μmoles light and long day conditions.

For *in vitro* culture, Arabidopsis seeds were surface-sterilized in 70% bleach, before transfer on agar plates containing half-strength salts and vitamins, 1.5% sucrose, and 0.8% agar, with or without kanamycin (50 μg/ml). Plants transferred to soil were grown under the same growth chamber conditions as mentioned above.

### Phylogenetic analysis

A region of 69 aminoacids spanning the highly conserved TCP box was used to build the phylogenetic trees. We used the sequences of the 24 *TCP* genes in Arabidopsis and the 25 genes in rice respectively. We followed the notation as in Martín-Trillo and Cubas (2010). For tomato we isolated the sequences from the ITAG 2.3 release of the sequenced tomato genome (http://solgenomics.net/organism/Solanum_lycopersicum/genome; Supplemental Table 1). The sequence alignments were made using Muscle (http://www.ebi.ac.uk/Tools/msa/muscle/). The tree was made using MEGA5 software (Tamura et al., 2011; http://www.megasoftware.net/) using the maximum likelihood method with 1000 bootstrap replicates.

### Construction of transgenes and plant transformation

To generate RNAi lines for At*TCP7*, a fragment of 195 bp of the coding sequence was amplified using primers M2LRNA2f (5′-TATCCGTGGAGCCACCAATT CTACTTCT-3′) and M2LRNA2r (5′-GCTGGTGTTGTCGTAAAGGTCTCATCTT-3′) and cloned into pCR8/GW/TOPO TA entry vector (Invitrogen) to generate pCR8-TCP7RNAi. As destination vector we used pK7GWIWG2(II) (Karimi et al., 2002).

For the GFP fusions with At*TCP7*, At*TCP15*, and At*TCP22*, we cloned the coding sequences of these genes into the entry vector pCR8/GW/TOPO TA and LR cloned into the plasmid pK2GW7 (Karimi et al., 2002) generating TCP7-OEX, TCP15-OEX, and TCP22-OEX, respectively (for primers used see Supplemental Table 4).

For the SRDX constructs, the coding sequence of At*TCP7* was cloned into the entry vector pCR8/GW/TOPO TA using forward primer M2Lf (5′-ATGTCTATTAACAACAACAACAACAACA-3′) and the reverse primer TCPSRDXrev (5′-TTACGCAAAGCCGAGGCGAAGTTCGAGATCAAGATCGAGACGTGGATCTTCCTCTCTTCGATCCGA-3′) without the stop codon of At*TCP7* and with the EAR peptide sequence LDLDLELRLGFA plus the terminator codon TAA (Hiratsu et al., 2003). The construct was LR cloned into the plasmid pK2GW7 generating TCP7-SRDX. Similarly, for At*TCP23* we used primers P23f (5′-ATGGAGTCCCACAACAACAACCAGAGCA-3′) and TCP23SRDXrev (5′-TTACGCAAAGCCGAGGCGAAGTTCGAGATCAAGATCGAGAGGAGAACCATCTATAGTAGGATTTTG-3′). The PCR fragment was cloned into pCR8/GW/TOPO TA and LR cloned in pK2GW7 to generate TCP23-SRDX.

To generate *Pro*At*TCP7:GUS, Pro*At*TCP8:GUS, Pro*At*TCP2:GUS*, and *Pro*At*TCP23:GUS* constructs, respectively, 1990, 1588, 2180, and 2494 bp of the complete 5′ intergenic region preceding the ORFs was amplified from Col-0 genomic DNA using primers indicated in Supplemental Table 4 and inserted into the binary vector pKGWFS7 (Karimi et al., 2002). Constructs were introduced into *Agrobacterium* strain GV3101 (pMP90) (Koncz and Schell, 1986) by electroporation. Plant transformation was done using the “floral dip” method (Clough and Bent, 1998).

### Subcellular localization analysis

To determine the cellular location of class I TCP factors, the GFP signal of roots in 50% glycerol of transgenic plants TCP7-OEX, TCP15-OEX, and TCP22-OEX was observed under an epifluorescence microscope (Nikon Eclipse E600, Nikon; excitation at 488 nm and emission at 495–575 nm).

### Gene expression analysis

Total RNA was extracted with RNeasy Plant Mini Kit (QIAGEN), and treated with on-column DNase (QIAGEN). cDNAs were generated with QuantiTect Reverse Transcription Kit (Qiagen). qPCR was performed on a MyiQ two-color real time PCR detection system with iQ SYBR Green supermix (Bio-Rad). Real-time PCR experiments were repeated using three biological replicates and one technical replicate. For tissue expression analysis of class I *TCP* genes the roots, young leaves, mature leaves and shoot apices were taken from 2-week old plants, the inflorescences were taken from the tip of the main stem. To analyze expression levels in mutant and transgenic backgrounds, three sets of 5–6 pooled entire 2-week old plants were collected and analyzed. *ACTIN8* was used as an endogenous control gene. The number of cycles for the RT-PCR was 28 for At*TCP7*, At*TCP8*, At*TCP14*, At*TCP15*, At*TCP21*, and At*TCP22*, 30 for At*TCP23* and 26 for *ACTIN8*. Primers used for RT-PCR and qPCR are specified in Supplemental Table 2.

### Construction of multiple mutants

To generate multiple *tcp* mutants, we made first the double mutants *tcp8-1 tcp15-1* and *tcp22-1 tcp23-1*. Double homozygous recessive plants were crossed and from F3 progenies we selected by genotyping the quadruple mutant *tcp8-1 tcp15-1 tcp22-1 tcp23-1*. This multiple mutant was crossed with *tcp21-1* and through selection by genotyping we isolated the pentuple mutant *tcp8-1 tcp15-1 tcp22-1 tcp23-1 tcp21-1*.

The primers used for the genotyping of the mutants are indicated in Supplemental Table 3. For *tcp8-1* we used the combination *tcp8-1*Lp and *tcp8-1*Rp for the wild type copy and *tcp8-1*Lp and LB1SAIL and *tcp8-1*Rp and LB1SAIL for the insertions. Similarly, for *tcp15-1*, we used the combinations *tcp15-1*Lp and *tcp15-1*Rp for the wild type copy and *tcp15-1*Lp and LBa1 for the mutation. For *tcp22-1*, we used primers *tcp22-1*Lp and *tcp22-1*Rp for the wild type copy and *tcp22-1*Rp and LBa1 for the insertion. For *tcp23-1*, we used the combinations *tcp23-1*Lp and *tcp23-1*Rp for the wild type copy and *tcp23-1*Lp and LB1SAIL and *tcp23-1*Rp and LB1SAIL for the insertions. For *tcp7-1*, we used the primers TCP7p1000 and M2LPROr for the wild type copy and TCP7p1000 and NS343_GK-LB and M2LPROr and NS344_GK-RB for the insertions. For *tcp7-2*, the primers NS256_S18Lp and NS257_S18Rp for the wild type copy and NS256_S18Lp and LB1SAIL and NS257_S18Rp and LB1SAIL for the insertions. For *tcp7-3*, the primers NS254_S17Lp and NS255_S17Rp for the wild type copy and NS255_S17Rp and NS290_RB4INRA for the mutant copy. In case of *tcp7-4*, the combinations NS254_S17Lp and NS255_S17Rp for the wild type copy and NS255_S17Rp and NS314_p745Wisc for the mutant copy. For *tcp21-1* or *che-1*, the primers NS550_CHEgF and NS551_CHEgR for the wild type copy and NS550_CHEgF and LBa1 for the insertion. For the design of the primers we used the web tool at http://signal.salk.edu/tdnaprimers.2.html. All the positions of the insertions were determined by sequencing.

### GUS histochemical assays

For histochemical GUS activity assays, plants were submerged in 90% acetone for 20 min. on ice, washed twice with 50mM sodium phosphate buffer (pH 7.2) and subsequently incubated in staining solution (0.1 M sodium phosphate buffer (pH 7.2), 0.5 mM Fe(CN)2, 0.5 mM Fe(CN)3, 0.1% Tween-20 and 2 mM 5-bromo-4-chloro-3-indolyl-β-d-glucuronide) at 37°C for 6 h. Plants were cleared and dehydrated through a graded ethanol series. For sections, FAA-fixed and dehydrated material was embedded in Paraplast Plus (McCormick Scientific, St. Louis, MO). The sections were dewaxed by immersion in Histoclear and mounted in Permount (Fisher Scientific).

### Yeast two-hybrid assay

For the yeast two-hybrid, the full-length coding sequences of At*TCP7*, At*TCP8*, At*TCP15*, At*TCP21*, At*TCP23*, At*TCP2*, and At*TCP3* were amplified by PCR using Pwo polymerase (Roche) and cloned into either pGBKT7 bait vector (fused with Gal4 DNA-binding domain; BD) or pGADT7 prey vector (fused with GAL4 activation domain; AD, Clontech, http://clontech.com). The constructs were verified by sequencing and co-transformed into yeast strain AH109. To test for interactions, the bait and prey constructs co-expressed in yeast were subjected to β–Galactosidase filter assay according to the manufacturer's instructions (Clontech).

### EMSA

We amplified the coding sequences of *AtTCP7, AtTCP15, AtTCP22*, and *AtTCP23*, and cloned them into the pCR8/GW/TOPO TA cloning vector (Invitrogen). We generated GST-fusion–protein-expressing constructs through LR reactions between the pCR8 entry clones and the destination vectors pDEST15 (Invitrogen) containing an N-terminal GST tag. All the fusion proteins and empty control plasmids were expressed in *Escherichia coli* strain BL21 (DE3).

The probe used in EMSA is 76 bp long, from −239 to −315 bp upstream of the starting codon of *STM* and was prepared by annealing the two biotin-labeled anti-parallel primers Kboxf 5′-AACTATCACTATTTAGAATTTTCAATGTGGAAAAGGAAGCTGATTGTTGAAGCATAAATCCCGGGAGACCACTTTT-3′ and Kboxr 5′-AAAAGTGGTCTCCCGGGATTTATGCTTCAACAATCAGCTTCCTTTTCCACATTGAAAATTCTAAATAGTGATAGTT-3′. The same two unlabeled primers were annealed for competition. For the EMSA, recombinant GST-tagged proteins on pDEST15 were purified on an immobilized glutathione column (Pierce). Binding reactions and detection of biotin-labeled probe was performed according to the manufacturer's instructions (LightShift Chemiluminescent EMSA Kit, Pierce).

### Phenotypic analysis

For the number of rosette leaves the plants were at bolting stage with 0.5 cm of the main stem elongated. We did not take account of the cotyledons. The petiole length, blade length, blade width, blade perimeter and blade area were determined using the sixth leaf of plants at bolting stage. The data were analyzed using LeafJ (Maloof et al., 2013) with ImageJ software (http://rsb.info.nih.gov/ij/). For leaf epidermis analysis, dental resin imprints and nail polish copies (Kagan et al., 1992) were taken from the medial leaf lamina zone from rosette leaves of Col wt, TCP7-SRDX, and TCP23-SRDX plants and analyzed by light microscopy in a Nikon Eclipse E600 (Nikon).

### Accession numbers

Sequence data from this article can be found in The Arabidopsis Information Resource (TAIR; http://www.arabidopsis.org/) or GenBank/EMBL databases under the following accession numbers: At*TCP7* (At5g23280), At*TCP8* (At1g58100), At*TCP22* (At1g72010), and At*TCP23* (At1g35560).

## Author contributions

José A. Aguilar-Martínez and Neelima Sinha designed the research. José A. Aguilar-Martínez performed the research. José A. Aguilar-Martínez and Neelima Sinha analyzed the data and wrote the paper.

## Supplementary material

Supplementary Figure 1 **Sequence alignment of the TCP box of class I TCP factors**.

Supplementary Table 1 **List of *TCP* genes in the tomato genome**.

Supplementary Table 2 **List of primers used in this study for gene expression analysis**.

Supplementary Table 3 **List of primers used to genotype *tcp* mutants in Arabidopsis**.

Supplementary Table 4 **List of primers for genetic constructs generated in this study**.
