# Supplementary material for: Analysis of the role of Arabidopsis class I TCP genes AtTCP7, AtTCP8, AtTCP22, and AtTCP23 in leaf development
Source: Front Plant Sci. 2013 Oct 16;4:406. doi: 10.3389/fpls.2013.00406 (PMC3797442; doi:10.3389/fpls.2013.00406)
Supplement: Supplementary Figure 1 — Sequence alignment of the TCP box of class I TCP factors. [file DataSheet1.DOC]

>TB1

KDRHSKICTAGGMRDRRMRLSLDVARKFFALQDMLGFDKASKTVQWLLNTSKSAIQEIMADDASSECVE----

>AtTCP16

KDRHLKIGG----RDRRIRIPPSVAPQLFRLTKELGFKTDGETVSWLLQNAEPAIFAATGHGVTTTSNEDIQP

>AtTCP6

KDRHLKVEG----RGRRVRLPPLCAARIYQLTKELGHKSDGETLEWLLQHAEPSILSATVNGIKPTESVVSQP

>AtTCP11

KDRHTKVNG----RSRRVTMPALAAARIFQLTRELGHKTEGETIEWLLSQAEPSIIAATGYGTKLISNWVDVA

>PCF1

SDRHSKVAG----RGRRVRIPAMVAARVFQLTRELGHRTDGETIEWLLRQAEPSIIAATGTGVTPEEAPPAAV

>SlTCP38

KDRHTKVNG----RGRRVRMPALCAARVFQLTKELGHRTDGETIEWLLRNAEPAIIAATGTGTVPATQVTTTS

>OsTCP25

RDRHVKVEG----RGRRIRMPVNCAARIAQLTRELGHKSDGETIRWLMQQSEPAIVAATGTGTVPAIATTVDG

>PCF2

RDRHTKVEG----RGRRIRMPAACAARIFQLTRELGHKSDGETIRWLLQQSEPAIIAATGTGTVPAIATTVDG

>SlTCP10

KDRHTKVEG----RGRRIRIPATCAARIFQLTRELGHKSDGETVRWLLEQAEQSIIEATGTGTVPAIAVSVNG

>AtTCP19

KDRHTKVEG----RGRRIRMPAGCAARVFQLTRELGHKSDGETIRWLLERAEPAIIEATGTGTVPAIAVSVNG

>AtTCP9

KDRHTKVEG----RGRRIRMPATCAARIFQLTRELGHKSDGETIRWLLENAEPAIIAATGTGTVPAIAMSVNG

>SlTCP20

KDRHTKVEG----RGRRIRMPAACAARIFQLTRELGHKSEGETIRWLLERAEPAIIAATGTGTVPAIAVSVNG

>SlTCP17

KDRHTKVEG----RGRRIRMPALCAARIFQLTRELGHKSDGETIQWLLQKAEPSIIAATGHGTIQASLYRRLD

>AtTCP20

KDRHTKVEG----RGRRIRMPALCAARIFQLTRELGHKSDGETIQWLLQQAEPSIIAATGSGTIPASALASSA

>SlTCP21

KDRHTKVEG----RGRRIRMPALCAARIFQLTRELGHKSDGETIQWLLQQAEPSIIAATGTGTIPASALAAAA

>OsTCP6

KDRHTKVDG----RGRRIRMPALCAARIFQLTRELGHKSDGETVQWLLQQAEPAIVAATGTGTIPASALASVA

>OsTCP7

KDRHSKVNG----RGRRVRMPIVCAARVFQLTRELGLKSDGQTIEWLLRQAEPSILAATGTGTTPAAFVSSSA

>OsTCP17

KDRHSKVNG----RGRRVRMPIVCAARVFQLTRELGLKSDGQTIEWLLRQAEPSILAATGSGTTPAVFSCSSA

>SlTCP8

KDRHSKVDG----RGRRIRMPIVCAARVFQLTRELGHKSDGQTIEWLLRQAEPSIIAATGTGTIPASFSTVSV

>SlTCP22

KDRHSKVDG----RGRRIRMPIVCAARVFQLTRELGHKSDGQTIEWLLRQAEPSIIAATGTGTIPASFSTVSV

>AtTCP7

KDRHSKVDG----RGRRIRMPIICAARVFQLTRELGHKSDGQTIEWLLRQAEPSIIAATGTGTTPASFSTASV

>AtTCP21

KDRHSKVDG----RGRRIRMPIICAARVFQLTRELGHKSDGQTIEWLLRQAEPSIIAATGTGTTPASFSTASL

>PCF3

KDRHTKVEG----RGRRIRMPALCAARVFQLTRELGHKTDGETIEWLLQQAEPAIVAATGTGTIPANFSSLAV

>AtTCP23

KDRHIKVDG----RGRRIRMPAICAARVFQLTRELQHKSDGETIEWLLQQAEPAIIAATGTGTIPANISTLNI

>SlTCP7

KDRHTKVDG----RGRRIRMPALCAARVFQLTKELGHKSDGETIEWLLQQAEPSIIAATGTGTIPANFSTLNV

>SlTCP23

KDRHTKVDG----RGRRIRMPALCAARVFQLTKELGHKSDGETIEWLLQQAEPSIIAATGTGTIPANFSTLNV

>AtTCP8

KDRHTKVDG----RGRRIRMPALCAARVFQLTRELGHKSDGETIEWLLQQAEPAIVAATGTGTIPANFSTLSV

>SlTCP9

KDRHTKVDG----RGRRIRMPALCAARVFQLTRELGHKSDGETIEWLLQQAEPAIIATTGTGTIPANFSTLNV

>AtTCP22

KDRHTKVDG----RGRRIRMPAMCAARVFQLTRELGHKSDGETIEWLLQQAEPAIIASTGTGTIPANFSTLNA

>SlTCP37

KDRHTKVDG----RGRRIRMPATCAARVFQLTRELGHKSDGETIEWLLQQAEPAVIAATGTGTIPANYSSLNI

>SlTCP24

KDRHTKVDG----RGRRIRMPAACAARVFQLTRELGHKSDGETIEWLLQQAEPAIIAATGTGTIPANFSTLNI

>OsTCP28

KDRHTKVDG----RGRRIRMPALCAARVFQLTRELGHKSDGETIEWLLQQAEPAIIAATGTGTIPANFSSLNI

>OsTCP19

KDRHTKVDG----RGRRIRMPAICAARVFQLTRELGHKTDGETIEWLLQQAEPAVIAATGTGTIPANFTSLNI

>AtTCP15

KDRHTKVEG----RGRRIRMPAMCAARVFQLTRELGHKSDGETIEWLLQQAEPAVIAATGTGTIPANFTSLNI

>OsTCP9

KDRHTKVEG----RGRRIRMPALCAARVFQLTRELGHKTDGETIEWLLQQAEPAVIAATGTGTIPANFTSLNI

>AtTCP14

KDRHTKVDG----RGRRIRMPALCAARVFQLTRELGHKSDGETIEWLLQQAEPSVIAATGTGTIPANFTSLNI

>SlTCP15

KDRHTKVDG----RGRRIRMPALCAARVFQLTRELGHKSDGETIEWLLQQAEPAVIAATGTGTIPANFTSLNI

>SlTCP16

KDRHTKVDG----RGRRIRMPALCAARVFQLTRELGHKSDGETIEWLLQQAEPAVIAATGTGTIPANFTSLNI

**Supplemental Fig. 1** Sequence alignment of the TCP box of class I TCP factors and maize TB1 using Muscle
