# Supplementary material for: Analysis of the role of Arabidopsis class I TCP genes AtTCP7, AtTCP8, AtTCP22, and AtTCP23 in leaf development
Source: Front Plant Sci. 2013 Oct 16;4:406. doi: 10.3389/fpls.2013.00406 (PMC3797442; doi:10.3389/fpls.2013.00406)
Supplement: Supplementary Table 1 — List of TCP genes in the tomato genome. [file DataSheet2.DOC]

**Supplemental Table 1.** List of tomato *TCP* genes, based on ITAG 2.3 release (<http://solgenomics.net/organism/Solanum_lycopersicum/genome>).

| **Gene name** | **Reference** | **Gene name** | **Reference** | **Gene name** | **Reference** |
| --- | --- | --- | --- | --- | --- |
| *SlTCP1* | Solyc04g006980.1.1 | *SlTCP14* | Solyc03g115010.1.1 | *SlTCP27* | Solyc02g065800.1.1 |
| *SlTCP2* | Solyc08g048390.1.1 | *SlTCP15* | Solyc06g070900.1.1 | *SlTCP28* | Solyc00g217560.1.1 |
| *SlTCP3* | Solyc08g048370.1.1 | *SlTCP16* | Solyc03g116320.1.1 | *SlTCP29* | Solyc02g077250.1.1 |
| *SlTCP4* | Solyc12g014140.1.1 | *SlTCP17* | Solyc02g094290.1.1 | *SlTCP30* | Solyc10g008780.1.1 |
| *SlTCP5* | Solyc07g062680.1.1 | *SlTCP18* | Solyc02g089020.1.1 | *SlTCP31* | Solyc11g045640.1.1 |
| *SlTCP6* | Solyc06g069460.1.1 | *SlTCP19* | Solyc03g119770.1.1 | *SlTCP32* | Solyc05g032780.1.1 |
| *SlTCP7* | Solyc06g065190.1.1 | *SlTCP20* | Solyc03g006800.1.1 | *SlTCP33* | Solyc06g069240.1.1 |
| *SlTCP8* | Solyc04g009180.1.1 | *SlTCP21* | Solyc02g068200.1.1 | *SlTCP34* | Solyc10g018710.1.1 |
| *SlTCP9* | Solyc11g020670.1.1 | *SlTCP22* | Solyc01g008230.1.1 | *SlTCP35* | Solyc03g045030.1.1 |
| *SlTCP10* | Solyc08g080150.1.1 | *SlTCP23* | Solyc06g065190.1.1 | *SlTCP36* | Solyc02g089830.1.1 |
| *SlTCP11* | Solyc07g053410.1.1 | *SlTCP24* | Solyc01g103780.1.1 | *SlTCP37* | Solyc05g007420.1.1 |
| *SlTCP12* | Solyc00g084870.1.1 | *SlTCP25* | Solyc02g089020.1.1 | *SlTCP38* | Solyc09g008030.1.1 |
| *SlTCP13* | Solyc05g009900.1.1 | *SlTCP26* | Solyc08g048370.1.1 |  |  |
