# Supplementary material for: Analysis of the role of Arabidopsis class I TCP genes AtTCP7, AtTCP8, AtTCP22, and AtTCP23 in leaf development
Source: Front Plant Sci. 2013 Oct 16;4:406. doi: 10.3389/fpls.2013.00406 (PMC3797442; doi:10.3389/fpls.2013.00406)
Supplement: Supplementary Table 2 — List of primers used in this study for gene expression analysis. [file DataSheet3.DOC]

**Supplemental Table 2.** Primers used for RT-PCR and qPCR.

| **Primer name** | **Sequence (5’-3’)** |
| --- | --- |
| TCP7RTPCRf | CAACAACAACAACAATAACAACGATGG |
| TCP7RTPCRr | AGCAAAGTCGATGGAAGAGGGAGAAG |
| TCP8RTPCRf | ATGGATCTCTCCGACATCCGAAACAACA |
| TCP8RTPCRr | TCACTCAGAGCTATTTGAGTTCTCCTCT |
| TCP14RTPCRf | ATGCAAAAGCCAACATCAAGTATCTTAAAT |
| TCP14RTPCRr | GATGGTTCAGCTTGTTGAAGAAGCCACTCT |
| TCP15RTPCRf | ATGGATCCGGATCCGGATCATAACCATC |
| TCP15RTPCRr | CTAGGAATGATGACTGGTGCTTCCATCT |
| NS315_TCP21cF | ATGGCCGACAACGACGGAGCAGTGAGTA |
| NS316_TCP21cR | TCAACGTGGTTCGTGGTCGTCTTCCCTC |
| TCP22RTPCRf | ATGAATCAGAATTCCTCTGTTGCGGAGG |
| TCP22RTPCRr | TCACTTTTTGTCATCACCACCATTTTCA |
| TCP23RTPCRf | ATGGAGTCCCACAACAACAACCAGAGCA |
| TCP23RTPCRr | TCAAGGAGAACCATCTATAGTAGGATTT |
| Act8RTPCRf | TAAACTAAAGAGACATCGTTTCCA |
| Act8RTPCRr | TTTTTATCCGAGTTTGAAGAGGC |
|  |  |
| NS1065_CYCA1;1F | AAAGCGATGGAGTTGAGAGG |
| NS1066_CYCA1;1R | GTGGGATTACGGATGGACAA |
| NS1061_CYCA2;3F | CCCAAGCCTTGAAGTCGAGTT |
| NS1062_CYCA2;3R | AAAACCGCTGAAGCAGCAAC |
| NS1041_qCLFF | GGCCACCAGATCGGAGCCACC |
| NS1042_qCLFR | GGTCTTCTATGGAACGAGGAG |
| NS726_STMQRTf | ATCATGGCTCATCCTCACTACC |
| NS727_STMQRTr | GACATCCTGTTGGTCCCATAGA |
| NS728_BPQRTf | TGGACTGCCAAAAGATTGGA |
| NS729_BPQRTr | GGCAGAGACAGACGGTGTTG |
| NS730_AS1QRTf | CAGAGGAAGAGCAGAGGCTTG |
| NS731_AS1QRTr | CTTCCCACCACTTCCCTAACC |
| NS732_ACT8QRTf | GGTAACATTGTGCTCAGTGGTGG |
| NS733_ACT8QRTr | AACGACCTTAATCTTCATGCTGC |
|  |  |
