# Supplementary material for: Analysis of the role of Arabidopsis class I TCP genes AtTCP7, AtTCP8, AtTCP22, and AtTCP23 in leaf development
Source: Front Plant Sci. 2013 Oct 16;4:406. doi: 10.3389/fpls.2013.00406 (PMC3797442; doi:10.3389/fpls.2013.00406)
Supplement: Supplementary Table 3 — List of primers used to genotype tcp mutants in Arabidopsis. [file DataSheet4.DOC]

**Supplemental Table 3.** Primers used for genotyping.

| **Primer name** | **Sequence (5’-3’)** |
| --- | --- |
| *tcp7-3*Lp | CACAATCTTTTTATAAAACTGCCG |
| *tcp7-3*Rp | GGCTGCTACGTGCATTTAAAG |
| *tcp7-5*Lp | TGGGGTCATTTAGATGATTCG |
| *tcp7-5*Rp | GGGAGATCGATTTCTTATCGG |
| NS254_S17Lp | TGGGGTCATTTAGATGATTCG |
| NS255_S17Rp | GGGAGATCGATTTCTTATCGG |
| NS256_S18Lp | CACAATCTTTTTATAAAACTGCCG |
| NS257_S18Rp | GGCTGCTACGTGCATTTAAAG |
| TCP7p1000 | cagtaaaacctctataaattaataac |
| M2LPROr | GGTTTTCGATCTGGGTTTAGTGTTTTCT |
| *tcp8-1*Lp | AAAAATACGCACGCTTCACTG |
| *tcp8-1*Rp | TTGAAGCTGATGATGAAACCC |
| *tcp15-1*Lp | AGAACCACGTAAGCCCATCTC |
| *tcp15-1*Rp | CACCACTACTCCAAAACGGTG |
| NS550_CHEgF | gctacttattttctacgaatcctaatt |
| NS551_CHEgR | TCCTCCGCTCTGACGACACGTTTCCCG |
| *tcp22-1*Lp | CGCATGAAGTACCAAGCTCTC |
| *tcp22-1*Rp | AATGTGGTGCCTCAACCTATG |
| *tcp23-1*Lp | TCAACACAAACTCATAGCCCC |
| *tcp23-1*Rp | TGAGGCTTTTGCTCAAGACTC |
| LBa1 | TGGTTCACGTAGTGGGCCATCG |
| LB1SAIL | GCCTTTTCAGAAATGGATAAATAGCC |
| NS314_p745Wisc | AACGTCCGCAATGTGTTATTAAGTTGTC |
| NS343_GK-LB | ATATTGACCATCATACTCATTGC |
| NS290_RB4INRA | TCACGGGTTGGGGTTTCTACAGGAC |
|  |  |
