# Supplementary material for: Analysis of the role of Arabidopsis class I TCP genes AtTCP7, AtTCP8, AtTCP22, and AtTCP23 in leaf development
Source: Front Plant Sci. 2013 Oct 16;4:406. doi: 10.3389/fpls.2013.00406 (PMC3797442; doi:10.3389/fpls.2013.00406)
Supplement: Supplementary Table 4 — List of primers for genetic constructs generated in this study. [file DataSheet5.DOC]

**Supplemental Table 4.** Primers used for genetic constructs.

| **Primer name** | **Sequence (5’-3’)** |
| --- | --- |
| M2Lf | ATGTCTATTAACAACAACAACAACAACA |
| M2Lr | TTAACGTGGATCTTCCTCTCTTCGATCC |
| P15f | ATGGATCCGGATCCGGATCATAACCATC |
| P15r | CTAGGAATGATGACTGGTGCTTCCATCT |
| P22Cf | ATGAATCAGAATTCCTCTGTTGCGGAGG |
| P22Cr | TCACTTTTTGTCATCACCACCATTTTCA |
|  |  |
| M2LPROf | AAACTTCTTGAACTAAAAATAATATAAA |
| M2LPROr | GGTTTTCGATCTGGGTTTAGTGTTTTCT |
| P8Prof | AGATCTGCACTTAATTTATATGCACATT |
| P8Pror | TTTCCGGTGAGAAAGAAGGGAAAAGAA |
| P22Fp | CTTCATTGTAGGGCCTTTTATAATTACC |
| P22Rp | CTTCAAATCCGTAAAAAGATATGATTTT |
| P23pf | ATAGACAAACAAACTCGGAACCAGCTTT |
| P23pr | GCTCTGGTTGTTGTTGTGGGACTCCAT |
|  |  |
